# Supplementary material for: Prevalence of diabetic retinopathy and vision-threatening diabetic retinopathy in adults with diabetes in China
Source: Nat Commun. 2023 Jul 18;14:4296. doi: 10.1038/s41467-023-39864-w (PMC10354077; doi:10.1038/s41467-023-39864-w)
Supplement: Supplementary file 1 — Supplementary Information File [file 41467_2023_39864_MOESM1_ESM.docx]

Supplementary Material

**Contents**

[1 Supplementary Tables for the Results Section 2](#_Toc138143923)

[1.1 Supplementary Table 1: Characteristics of study participants by DR severity. 2](#_Toc138143924)

[1.2 Supplementary Table 2: Comparison of characteristics of Northerners and Southerners. 5](#_Toc138143925)

[2 Supplementary Tables for the Methods Section 7](#_Toc138143926)

[2.1 Supplementary Table 3: General characteristics of study participants with gradable and ungradable fundus photos. 7](#_Toc138143927)

[2.2 Supplementary Table 4: Investigation period of 488 neighborhoods or rural villages of 122 study sites across 31 provinces. 8](#_Toc138143928)

[2.3 Supplementary Table 5: The sex-, age-, and rural/urban structure of Chinese adults with diabetes aged 18-74 in China in 2018-2019. 28](#_Toc138143929)

[3 Supplementary Table for the Study Group 29](#_Toc138143930)

[3.1 Supplementary Table 6: China National Diabetic Chronic Complications (DiaChronic) Study Group 29](#_Toc138143931)

# **1 Supplementary Tables for the Results Section**

# **1.****1 Supplementary Table 1: Characteristics of study participants by DR severity.**

|  | **Total (n=50564)** | **No DR (n=42005)** | **Any DR (n=8559)** |  | **p for linear trend^*^** |
| --- | --- | --- | --- | --- | --- |
|  |  |  | **Non-VTDR (n=6886)** | **VTDR (n=1673)** |  |
| **Demographics** |  |  |  |  |  |
| Female | 25448 (50.3) | 21284 (50.7) | 3353 (48.7) | 811 (48.5) | 0.0010 |
| Age, y | 57.5 (50.9-64.8) | 57.4 (50.7-64.9) | 58.1 (52.0-65.0) | 56.7 (51.2-64.0) | 0.0002 |
| **Geographic region** |  |  |  |  |  |
| North^†^ | 24862 (49.2) | 20106 (47.9) | 3769 (54.7) | 987 (59.0) | <0.0001 |
| **Setting** |  |  |  |  |  |
| Rural | 23639 (46.8) | 19427 (46.2) | 3388 (49.2) | 824 (49.3) | <0.0001 |
| **Socio-economic status** |  |  |  |  |  |
| High school and above | 12946 (25.6) | 11089 (26.4) | 1513 (22.0) | 344 (20.6) | <0.0001 |
| Average annual household income per capita |  |  |  |  | <0.0001 |
| <¥10000 | 14507 (28.7) | 11769 (28.0) | 2166 (31.5) | 572 (34.2) |  |
| ¥10000-<¥20000 | 10049 (19.9) | 8317 (19.8) | 1390 (20.2) | 342 (20.4) |  |
| ≥¥20000 | 13877 (27.4) | 11878 (28.3) | 1632 (23.7) | 367 (21.9) |  |
| Unwilling to disclose | 12131 (24.0) | 10041 (23.9) | 1698 (24.7) | 392 (23.4) |  |
| **Clinical characteristics**^‡^ |  |  |  |  |  |
| History of DR | 3033 (6.0) | 1844 (4.4) | 642 (9.4) | 547 (32.8) | <0.0001 |
| Diabetes duration, y | 5.2 (2.5-10.1) | 4.9 (2.3-9.2) | 7.9 (4.0-12.8) | 10.6 (5.9-16.3) | <0.0001 |
| Family history of diabetes | 20766 (41.1) | 16901 (40.2) | 3057 (44.4) | 808 (48.3) | <0.0001 |
| BMI, kg/m^2^ | 25.5 (23.3-27.8) | 25.5 (23.3-27.9) | 25.3 (23.1-27.7) | 25.1 (22.9-27.5) | <0.0001 |
| FPG, mmol/L | 8.06 (6.55-10.41) | 7.85 (6.43-9.96) | 9.37 (7.4-12.41) | 10.00 (7.76-13.00) | <0.0001 |
| HbA1c, % | 7.2 (6.2-8.6) | 7.0 (6.1-8.4) | 8.1 (6.9-9.7) | 8.5 (7.3-9.9) | <0.0001 |
| SBP, mmHg | 135.3 (123.0-149.3) | 134.3 (122.7-148.0) | 139.0 (125.7-154.0) | 140.3 (126.7-156.3) | <0.0001 |
| DBP, mmHg | 79.7 (72.7-87.0) | 79.7 (72.7-87.0) | 80.0 (72.7-87.7) | 79.7 (72.0-87.7) | 0.0101 |
| HDL-C, mmol/L | 1.21 (1.01-1.46) | 1.21 (1.01-1.46) | 1.22 (1.01-1.47) | 1.22 (1.01-1.48) | 0.0099 |
| LDL-C, mmol/L | 2.96 (2.33-3.61) | 2.95 (2.32-3.59) | 2.98 (2.36-3.67) | 3.07 (2.42-3.76) | <0.0001 |
| TG, mmol/L | 1.76 (1.22-2.66) | 1.77 (1.23-2.67) | 1.73 (1.19-2.63) | 1.74 (1.21-2.69) | 0.0178 |
| **Medications**^§^ |  |  |  |  |  |
| Glucose-lowering treatment | 39733 (78.6) | 32313 (76.9) | 5937 (86.2) | 1483 (88.6) | <0.0001 |
| Antihypertensive treatment | 19755 (39.1) | 16331 (38.9) | 2737 (39.7) | 687 (41.1) | 0.1063 |
| Lipid-lowering treatment | 6236 (12.3) | 5214 (12.4) | 827 (12.0) | 195 (11.7) | 0.1544 |
| **Attainment of targets** |  |  |  |  |  |
| HbA1c <7.0 % | 22229 (44.0) | 20156 (48.0) | 1753 (25.5) | 320 (19.1) | <0.0001 |
| BP <130/80 mmHg | 15044 (29.8) | 12842 (30.6) | 1791 (26.0) | 411 (24.6) | <0.0001 |
| LDL-C <2.6 mmol/L | 17628 (34.9) | 14790 (35.3) | 2310 (33.6) | 528 (31.6) | <0.0001 |
| **Lifestyle factors** |  |  |  |  |  |
| Physical activity ≥600 MET minutes/week^\|\|^ | 39777 (78.7) | 33243 (79.1) | 5275 (76.6) | 1259 (75.3) | <0.0001 |
| Current smoker | 11698 (23.1) | 9787 (23.3) | 1600 (23.2) | 311 (18.6) | <0.0001 |
| Current drinker | 13811 (27.3) | 11628 (27.7) | 1844 (26.8) | 339 (20.3) | <0.0001 |
| Dietary intake |  |  |  |  |  |
| Refined grains, g/day | 300 (160-450) | 300 (150-450) | 300 (180-450) | 300 (150-400) | 0.5483 |
| Whole grains, g/day | 14.3 (1.7-50.0) | 14.3 (1.7-50.0) | 14.3 (1.7-50.0) | 14.3 (1.7-57.1) | 0.0958 |
| Potatoes, g/day | 11.4 (1.7-30.8) | 12.9 (1.7-34.3) | 8.6 (0.7-28.6) | 7.1 (0.3-28.6) | <0.0001 |
| Soybean products, g/day | 14.3 (3.3-30.0) | 14.3 (3.3-30.0) | 14.3 (3.3-30.0) | 13.3 (2.3-28.6) | 0.0367 |
| Fresh vegetables, g/day | 300 (150-500) | 300 (150-500) | 300 (150-500) | 300 (150-500) | 0.0492 |
| Fresh fruits, g/day | 28.6 (3.3-100.0) | 28.6 (3.3-100.0) | 21.4 (0.8-80.0) | 14.3 (0.0-60.0) | <0.0001 |
| Dairy products, ml/day | 1.4 (0.0-100.0) | 1.4 (0.0-100.0) | 0.0 (0.0-85.7) | 2.7 (0.0-100.0) | 0.3052 |
| Red meat, g/day | 42.9 (14.3-100.0) | 42.9 (14.3-100.0) | 40.0 (12.9-100.0) | 34.3 (8.6-85.7) | 0.0010 |
| Poultry, g/day | 6.7 (0.9-15.0) | 6.7 (1.0-15.7) | 5.0 (0.8-14.3) | 3.3 (0.3-14.3) | 0.0061 |
| Seafood, g/day | 6.7 (0.8-25.7) | 7.1 (0.8-28.6) | 6.7 (0.5-21.4) | 5.0 (0.3-17.1) | 0.4571 |
| Eggs, g/day | 25.7 (7.9-50.0) | 25.7 (7.9-50.0) | 24.1 (7.3-50.0) | 28.6 (7.9-50.0) | 0.8104 |
| Nuts >0 g/day | 34309 (67.9) | 28741 (68.4) | 4511 (65.5) | 1057 (63.2) | <0.0001 |
| Fresh juices >0 ml/day | 3464 (6.9) | 2977 (7.1) | 397 (5.8) | 90 (5.4) | <0.0001 |

Any DR=any diabetic retinopathy. VTDR=vision-threatening diabetic retinopathy. BMI=body-mass index. FPG=fasting plasma glucose. HbA1c=hemoglobin A1c. SBP=systolic blood pressure. DBP=diastolic blood pressure. HDL-C=high-density lipoprotein cholesterol. LDL-C=low-density lipoprotein cholesterol.TG=triglycerides. BP=blood pressure. MET=metabolic equivalent.

Data were presented as median (25th percentile-75th percentile) for continuous variables or number (percentage) for categorical variables.

^*^ p value for linear trend was calculated using the two-sided linear regression test for continuous variables and the two-sided logistic regression test for categorical variables after adjustment for gender and age except gender only adjusted for age and age group only adjusted for gender.

^†^ The Northern region includes Beijing, Tianjin, Hebei, Shanxi, Inner Mongolia, Liaoning, Jilin, Heilongjiang, Shandong, Henan, Shaanxi, Gansu, Qinghai, Ningxia, and Xinjiang; the Southern region includes Shanghai, Jiangsu, Zhejiang, Anhui, Fujian, Jiangxi, Hubei, Hunan, Guangdong, Guangxi, Hainan, Chongqing, Sichuan, Guizhou, Yunnan, and Tibet.

^‡^ There were 45, 27, 117, 117, and 117 missing values for FPG, HbA1c, HDL-C, LDL-C, and TG, respectively. In addition, there were 196 missing values for history of DR, 12 for body-mass index, and 30 for blood pressure.

^§^ Antihypertensive treatment included angiotensin-converting enzyme inhibitor, angiotensin receptor blocker, aldosterone, β-blocker, α-blocker, diuretic, calcium antagonist, and others. Lipid-lowering treatment included statin, fibrate, and others.

^||^ METs were calculated according to a total of moderate- and vigorous-intensity physical activities (moderate MET value was equal to 4.0, and vigorous MET value was equal to 8.0) for work, in-transit, and leisure time throughout a week.

# **1.****2 Supplementary Table 2: Comparison of characteristics of Northerners and Southerners.**

|  | **Northerners**^*^ **(n=24862)** | **Southerners**^*^ **(n=25702)** | **P**^†^ |
| --- | --- | --- | --- |
| **Demographic** |  |  |  |
| Female | 12473 (50.2) | 12975 (50.5) | 0.4809 |
| Age, y | 57.6 (50.5-64.7) | 57.4 (51.2-65.0) | 0.0001 |
| **Setting** |  |  |  |
| Rural | 11995 (48.2) | 11644 (45.3) | <0.0001 |
| **Socio-economic characteristics** |  |  |  |
| High school and above | 6956 (28.0) | 5990 (23.3) | <0.0001 |
| Average annual household income per capita |  |  | <0.0001 |
| <¥10000 | 8212 (33.0) | 6295 (24.5) |  |
| ¥10000-<¥20000 | 5429 (21.8) | 4620 (18.0) |  |
| ≥¥20000 | 6393 (25.7) | 7484 (29.1) |  |
| Unwilling to disclose | 4828 (19.4) | 7303 (28.4) |  |
| **Clinical characteristics**^‡^ |  |  |  |
| History of DR | 1630 (6.6) | 1403 (5.5) | <0.0001 |
| Diabetes duration, y | 5.3 (2.4-10.2) | 5.2 (2.5-9.9) | 0.0012 |
| Family history of diabetes | 6956 (28.0) | 5990 (23.3) | <0.0001 |
| BMI, kg/m^2^ | 26.0 (23.8-28.4) | 25.0 (22.8-27.3) | <0.0001 |
| FPG, mmol/L | 8.04 (6.48-10.36) | 8.09 (6.62-10.46) | <0.0001 |
| HbA1c, % | 7.2 (6.2-8.6) | 7.2 (6.3-8.6) | 0.0920 |
| SBP, mmHg | 135.3 (123.3-149.3) | 135.0 (122.7-149.3) | 0.0099 |
| DBP, mmHg | 80.3 (73.0-87.3) | 79.3 (72.0-86.7) | <0.0001 |
| HDL-C, mmol/L | 1.18 (0.99-1.42) | 1.24 (1.03-1.50) | <0.0001 |
| LDL-C, mmol/L | 2.93 (2.31-3.56) | 2.99 (2.35-3.65) | <0.0001 |
| TG, mmol/L | 1.80 (1.26-2.70) | 1.73 (1.19-2.63) | <0.0001 |
| **Medications^§^** |  |  |  |
| Glucose-lowering treatment | 19247 (77.4) | 20486 (79.7) | <0.0001 |
| Antihypertensive treatment | 10168 (40.9) | 9587 (37.3) | <0.0001 |
| Lipid-lowering treatment | 3526 (14.2) | 2710 (10.5) | <0.0001 |
| **Attainment of targets** |  |  |  |
| HbA1c <7.0 % | 10814 (43.5) | 11415 (44.4) | 0.0328 |
| BP <130/80 mmHg | 7144 (28.7) | 7900 (30.8) | <0.0001 |
| LDL-C <2.6 mmol/L | 8935 (36.0) | 8693 (33.9) | <0.0001 |
| **Lifestyles** |  |  |  |
| Physical activity ≥600 MET minutes/week^\|\|^ | 19461 (78.3) | 20316 (79.0) | 0.0350 |
| Current smoker | 5798 (23.3) | 5900 (23.0) | 0.3301 |
| Current drinker | 7024 (28.3) | 6787 (26.4) | <0.0001 |
| Dietary intake |  |  |  |
| Refined grains, g/day | 300 (160-450) | 300 (150-400) | <0.0001 |
| Whole grains, g/day | 28.6 (6.7-64.3) | 6.7 (0.0-28.6) | <0.0001 |
| Potatoes, g/day | 14.3 (3.3-42.9) | 7.1 (0.8-28.0) | <0.0001 |
| Soybean products, g/day | 15.0 (5.7-42.9) | 10.0 (2.7-28.6) | <0.0001 |
| Fresh vegetables, g/day | 300 (150-450) | 300 (160-500) | <0.0001 |
| Fresh fruits, g/day | 28.6 (2.7-100.0) | 25.7 (3.3-80.0) | <0.0001 |
| Dairy products, ml/day | 6.7 (0.0-107.1) | 0.0 (0.0-71.4) | <0.0001 |
| Red meat, g/day | 28.6 (7.1-60.0) | 50.0 (21.4-100.0) | <0.0001 |
| Poultry, g/day | 3.3 (0.3-14.3) | 7.1 (1.7-21.4) | <0.0001 |
| Seafood, g/day | 3.3 (0.0-14.3) | 14.3 (3.3-35.7) | <0.0001 |
| Eggs, g/day | 34.3 (14.3-55.0) | 21.4 (7.1-50.0) | <0.0001 |
| Nuts >0g/day | 16716 (67.2) | 17593 (68.4) | 0.0035 |
| Fresh juices >0ml/day | 1633 (6.6) | 1831 (7.1) | 0.0134 |

BMI=body-mass index. FPG=fasting plasma glucose. HbA1c=hemoglobin A1c. SBP=systolic blood pressure. DBP=diastolic blood pressure. HDL-C=high-density lipoprotein cholesterol. LDL-C=low-density lipoprotein cholesterol.TG=triglycerides. BP=blood pressure. MET=metabolic equivalent.

Data were presented as median (interquartile ranges) for continuous variables or number (percentage) for categorical variables.

^*^ The Northern region includes Beijing, Tianjin, Hebei, Shanxi, Inner Mongolia, Liaoning, Jilin, Heilongjiang, Shandong, Henan, Shaanxi, Gansu, Qinghai, Ningxia, and Xinjiang; the Southern region includes Shanghai, Jiangsu, Zhejiang, Anhui, Fujian, Jiangxi, Hubei, Hunan, Guangdong, Guangxi, Hainan, Chongqing, Sichuan, Guizhou, Yunnan, and Tibet.

^†^ p was calculated using the two-sided Wilcoxon rank test for continuous variables, and the two-sided chi-square test for categorical variables.

^‡^ There were 45, 27, 117, 117, and 117 missing values for FPG, HbA1c, HDL-C, LDL-C, and TG, respectively. In addition, there were 12 missing values for body-mass index and 30 for blood pressure.

^§^ Antihypertensive treatment included angiotensin-converting enzyme inhibitor, angiotensin receptor blocker, aldosterone, β-blocker, α-blocker, diuretic, calcium antagonist, and others. Lipid-lowering treatment included statin, fibrate, and others.

^||^ METs were calculated according to a total of moderate- and vigorous-intensity physical activities (moderate MET value was equal to 4.0, and vigorous MET value was equal to 8.0) for work, in-transit, and leisure time throughout a week.

# **2 Supplementary Tables for the Methods Section**

# **2.1 Supplementary Table 3: General characteristics of study participants with gradable and ungradable fundus photos.**

|  | **Ungradable (n=1570)** | **Gradable (n=50564)** | **p**^*^ |
| --- | --- | --- | --- |
| **Demographic characteristics** |  |  |  |
| Female | 802 (51.1) | 25448 (50.3) | 0.5560 |
| Age, y | 63.7 (55.3-68.7) | 57.5 (50.9-64.8) | <0.0001 |
| **Socio-economic characteristic** |  |  |  |
| High school and above | 283 (18.0) | 12946 (25.6) | <0.0001 |
| **Clinical characteristics**^†^ |  |  |  |
| Diabetes duration, y | 8.7 (4.3-15.1) | 5.2 (2.5-10.1) | <0.0001 |
| HbA1c, % | 8.0 (6.7-9.5) | 7.2 (6.2-8.6) | <0.0001 |
| SBP, mmHg | 140.0 (125.7-154.7) | 135.3 (123.0-149.3) | <0.0001 |
| DBP, mmHg | 78.0 (70.0-86.0) | 79.7 (72.7-87.0) | <0.0001 |
| HDL-C, mmol/L | 1.25 (1.03-1.51) | 1.21 (1.01-1.46) | 0.0001 |
| LDL-C, mmol/L | 2.98 (2.36-3.65) | 2.96 (2.33-3.61) | 0.3717 |
| TG, mmol/L | 1.75 (1.22-2.52) | 1.76 (1.22-2.66) | 0.0871 |

HbA1c=hemoglobin A1c. SBP=systolic blood pressure. DBP=diastolic blood pressure. HDL-C=high-density lipoprotein cholesterol. LDL-C=low-density lipoprotein cholesterol.TG=triglycerides. BP=blood pressure.

Data were presented as median (interquartile range) for continuous variables and number (percentage) for categorical variables.

^*^ p was calculated using the Wilcoxon rank test for continuous variables, and the chi-square test for categorical variables.

^†^ There were 30, 126, 126, and 126 missing values for HbA1c, HDL-C, LDL-C, and TG, respectively. In addition, there were 31 missing values for blood pressure.

# **2.2 Supplementary Table 4: Investigation period of 488 neighborhoods or rural villages of 122 study sites across 31 provinces.**

| **Province** | | **Study site** | | **Setting** | **Neighborhoods/villages** | | **Period of Investigation** |
| --- | --- | --- | --- | --- | --- | --- | --- |
| **Name** | **No.** | **Name** | **No.** |  | **Name** | **No.** |  |
| Beijing | 1 | Dongcheng District | 1 | Urban | Dongsi Community | 1 | 2018/06/20-2018/06/27 |
| Beijing | 1 | Dongcheng District | 1 | Urban | Hepingli Subdistrict | 2 |  |
| Beijing | 1 | Dongcheng District | 1 | Urban | Beixinqiao | 3 |  |
| Beijing | 1 | Dongcheng District | 1 | Urban | Dongzhimen Subdistrict | 4 |  |
| Beijing | 1 | Tongzhou District | 2 | Urban | Liyuan Town | 5 | 2018/06/06-2018/06/19 |
| Beijing | 1 | Tongzhou District | 2 | Urban | Yongledian Town | 6 |  |
| Beijing | 1 | Tongzhou District | 2 | Urban | Lucheng Town | 7 |  |
| Beijing | 1 | Tongzhou District | 2 | Urban | Yujiawu | 8 |  |
| Beijing | 1 | Changping District | 3 | Urban | Tiannan Subdistrict | 9 | 2018/06/29-2018/07/06 |
| Beijing | 1 | Changping District | 3 | Urban | Nankou | 10 |  |
| Beijing | 1 | Changping District | 3 | Urban | Machikou | 11 |  |
| Beijing | 1 | Changping District | 3 | Urban | Baishan | 12 |  |
| Beijing | 1 | Huairou District | 4 | Urban | Quanhe Subdistrict | 13 | 2018/05/24-2018/05/31 |
| Beijing | 1 | Huairou District | 4 | Urban | Huairou Town | 14 |  |
| Beijing | 1 | Huairou District | 4 | Urban | Miaocheng Town | 15 |  |
| Beijing | 1 | Huairou District | 4 | Urban | Yanqi Town | 16 |  |
| Tianjin | 2 | Hexi District | 5 | Urban | Jianshan Street | 17 | 2019/12/09-2019/12/13 |
| Tianjin | 2 | Hexi District | 5 | Urban | Chentangzhuang Street | 18 |  |
| Tianjin | 2 | Hexi District | 5 | Urban | Xiawafang Street | 19 |  |
| Tianjin | 2 | Hexi District | 5 | Urban | Liulin Street | 20 |  |
| Tianjin | 2 | Nankai District | 6 | Urban | Water Park Street | 21 | 2019/08/17-2019/08/25 |
| Tianjin | 2 | Nankai District | 6 | Urban | Xingnan Street | 22 |  |
| Tianjin | 2 | Nankai District | 6 | Urban | Jialing Road Street | 23 |  |
| Tianjin | 2 | Nankai District | 6 | Urban | Wanxing Street | 24 |  |
| Tianjin | 2 | Jinnan District | 7 | Urban | Beizhakou Town | 25 | 2019/09/17-2019/09/28 |
| Tianjin | 2 | Jinnan District | 7 | Urban | Xianshuigu Town | 26 |  |
| Tianjin | 2 | Jinnan District | 7 | Urban | Balitai (Shuangzha) | 27 |  |
| Tianjin | 2 | Jinnan District | 7 | Urban | Balitai Town | 28 |  |
| Tianjin | 2 | Ji County | 8 | Urban | Sangzi Town | 29 | 2019/08/31-2019/09/04 |
| Tianjin | 2 | Ji County | 8 | Urban | Chutouling Town | 30 |  |
| Tianjin | 2 | Ji County | 8 | Urban | Limingzhuang Town | 31 |  |
| Tianjin | 2 | Ji County | 8 | Urban | Xiawotou Town | 32 |  |
| Hebei | 3 | Xinhua District, Shijiazhuang City | 9 | Urban | Lianmeng Subdistrict | 33 | 2019/05/06-2019/05/12 |
| Hebei | 3 | Xinhua District, Shijiazhuang City | 9 | Urban | Hezuo Road Subdistrict | 34 |  |
| Hebei | 3 | Xinhua District, Shijiazhuang City | 9 | Urban | Dongjiao Subdistrict | 35 |  |
| Hebei | 3 | Xinhua District, Shijiazhuang City | 9 | Urban | Tianyuan Subdistrict | 36 |  |
| Hebei | 3 | Qian'an City | 10 | Rural | Caiyuan Town | 37 | 2019/06/24-2019/06/30 |
| Hebei | 3 | Qian'an City | 10 | Rural | Yanjiadian Township | 38 |  |
| Hebei | 3 | Qian'an City | 10 | Rural | Shangzhuang Township | 39 |  |
| Hebei | 3 | Qian'an City | 10 | Rural | Shaheyi Town | 40 |  |
| Hebei | 3 | Xingtai County | 11 | Rural | Huangsi Town | 41 | 2019/05/15-2019/05/30 |
| Hebei | 3 | Xingtai County | 11 | Rural | Huining Town | 42 |  |
| Hebei | 3 | Xingtai County | 11 | Rural | Nanshimen Town | 43 |  |
| Hebei | 3 | Xingtai County | 11 | Rural | Yanjiatun Town | 44 |  |
| Hebei | 3 | Sanhe City | 12 | Rural | Yanjiao Development Zone | 45 | 2019/06/16-2019/06/23 |
| Hebei | 3 | Sanhe City | 12 | Rural | Huangtuzhuang Town | 46 |  |
| Hebei | 3 | Sanhe City | 12 | Rural | Liqizhuang | 47 |  |
| Hebei | 3 | Sanhe City | 12 | Rural | Xinji Town | 48 |  |
| Shanxi | 4 | Xinghualing District | 13 | Urban | Sanqiao Subdistrict | 49 | 2019/07/19-2019/07/26 |
| Shanxi | 4 | Xinghualing District | 13 | Urban | Jianhe Subdistrict | 50 |  |
| Shanxi | 4 | Xinghualing District | 13 | Urban | Balingqiao Subdistrict | 51 |  |
| Shanxi | 4 | Xinghualing District | 13 | Urban | Xinghualing Subdistrict | 52 |  |
| Shanxi | 4 | Huguan County | 14 | Rural | Jidian Township | 53 | 2019/06/18-2019/06/25 |
| Shanxi | 4 | Huguan County | 14 | Rural | Jinzhuang Town | 54 |  |
| Shanxi | 4 | Huguan County | 14 | Rural | Longquan Town | 55 |  |
| Shanxi | 4 | Huguan County | 14 | Rural | Shuzhang Town | 56 |  |
| Shanxi | 4 | Yuci District, Jinzhong City | 15 | Urban | Guojiabao Township | 57 | 2019/07/09-2019/07/16 |
| Shanxi | 4 | Yuci District, Jinzhong City | 15 | Urban | Wujin Mountain Town | 58 |  |
| Shanxi | 4 | Yuci District, Jinzhong City | 15 | Urban | Zhuangzi Township | 59 |  |
| Shanxi | 4 | Yuci District, Jinzhong City | 15 | Urban | Beiguan Subdistrict | 60 |  |
| Shanxi | 4 | Jiang County | 16 | Rural | Gujiang Town | 61 | 2019/06/28-2019/07/05 |
| Shanxi | 4 | Jiang County | 16 | Rural | Chencun Town | 62 |  |
| Shanxi | 4 | Jiang County | 16 | Rural | Hengshui Town | 63 |  |
| Shanxi | 4 | Jiang County | 16 | Rural | Haozhuang Township | 64 |  |
| Inner Mongolia | 5 | Kailu County | 17 | Rural | Bao'an Farm | 65 | 2018/11/13-2018/12/12 |
| Inner Mongolia | 5 | Kailu County | 17 | Rural | Maixin Town | 66 |  |
| Inner Mongolia | 5 | Kailu County | 17 | Rural | Jianhua Town | 67 |  |
| Inner Mongolia | 5 | Kailu County | 17 | Rural | Kailu Town | 68 |  |
| Inner Mongolia | 5 | Ordos City Ejin Holo Banner | 18 | Rural | A Town | 69 | 2018/11/28-2018/12/17 |
| Inner Mongolia | 5 | Ordos City Ejin Holo Banner | 18 | Rural | Wulanmulun Town | 70 |  |
| Inner Mongolia | 5 | Ordos City Ejin Holo Banner | 18 | Rural | Nalintaohai Town | 71 |  |
| Inner Mongolia | 5 | Ordos City Ejin Holo Banner | 18 | Rural | Ejin horo Town | 72 |  |
| Inner Mongolia | 5 | Yakeshi City | 19 | Rural | Wu'er Qihan Town | 73 | 2018/10/23-2018/10/30 |
| Inner Mongolia | 5 | Yakeshi City | 19 | Rural | Nuanquan | 74 |  |
| Inner Mongolia | 5 | Yakeshi City | 19 | Rural | Shengli | 75 |  |
| Inner Mongolia | 5 | Yakeshi City | 19 | Rural | Yongxing | 76 |  |
| Inner Mongolia | 5 | Linhe District, Bayannaoer City | 20 | Urban | Xianfeng | 77 | 2018/10/09-2018/10/15 |
| Inner Mongolia | 5 | Linhe District, Bayannaoer City | 20 | Urban | Bainaobao Town | 78 |  |
| Inner Mongolia | 5 | Linhe District, Bayannaoer City | 20 | Urban | Ganzhaomiao Town | 79 |  |
| Inner Mongolia | 5 | Linhe District, Bayannaoer City | 20 | Urban | Xihuan Road Subdistrict | 80 |  |
| Liaoning | 6 | Shahekou District | 21 | Urban | Zhongshan Park | 81 | 2019/03/25-2019/04/12 |
| Liaoning | 6 | Shahekou District | 21 | Urban | Xinggong Subdistrict | 82 |  |
| Liaoning | 6 | Shahekou District | 21 | Urban | Lijia Subdistrict | 83 |  |
| Liaoning | 6 | Shahekou District | 21 | Urban | Heishijiao | 84 |  |
| Liaoning | 6 | Tiexi District, Anshan City | 22 | Urban | Bajiazi Subdistrict | 85 | 2018/10/10-2018/10/26 |
| Liaoning | 6 | Tiexi District, Anshan City | 22 | Urban | Xingsheng Subdistrict | 86 |  |
| Liaoning | 6 | Tiexi District, Anshan City | 22 | Urban | Beitao Subdistrict | 87 |  |
| Liaoning | 6 | Tiexi District, Anshan City | 22 | Urban | Dalu Subdistrict | 88 |  |
| Liaoning | 6 | Qingyuan Manchu Autonomous County | 23 | Rural | Wandianzi Town | 89 | 2018/11/22-2019/03/29 |
| Liaoning | 6 | Qingyuan Manchu Autonomous County | 23 | Rural | Xiajiabao Town | 90 |  |
| Liaoning | 6 | Qingyuan Manchu Autonomous County | 23 | Rural | Gounaidian Township | 91 |  |
| Liaoning | 6 | Qingyuan Manchu Autonomous County | 23 | Rural | Nanshancheng Town | 92 |  |
| Liaoning | 6 | Dawa District | 24 | Urban | Dongfeng Town | 93 | 2018/12/18-2019/01/04 |
| Liaoning | 6 | Dawa District | 24 | Urban | Yushu Subdistrict | 94 |  |
| Liaoning | 6 | Dawa District | 24 | Urban | Xinli Town | 95 |  |
| Liaoning | 6 | Dawa District | 24 | Urban | Tangjia Town | 96 |  |
| Jilin | 7 | Dehui City, Changchun City | 25 | Rural | Daqingju Town | 97 | 2018/06/05-2018/07/02 |
| Jilin | 7 | Dehui City, Changchun City | 25 | Rural | Guojia Town | 98 |  |
| Jilin | 7 | Dehui City, Changchun City | 25 | Rural | Tiantai Town | 99 |  |
| Jilin | 7 | Dehui City, Changchun City | 25 | Rural | Biangang Township | 100 |  |
| Jilin | 7 | Fengman District | 26 | Urban | Gaoxin Subdistrict | 101 | 2018/08/22-2018/10/11 |
| Jilin | 7 | Fengman District | 26 | Urban | Wangqi Town | 102 |  |
| Jilin | 7 | Fengman District | 26 | Urban | Hongqi Subdistrict | 103 |  |
| Jilin | 7 | Fengman District | 26 | Urban | Xiaobaishan Township | 104 |  |
| Jilin | 7 | Ji'an City | 27 | Rural | Tuanjie Subdistrict | 105 | 2018/06/25-2018/07/09 |
| Jilin | 7 | Ji'an City | 27 | Rural | Liming Subdistrict | 106 |  |
| Jilin | 7 | Ji'an City | 27 | Rural | Huadian Town | 107 |  |
| Jilin | 7 | Ji'an City | 27 | Rural | Qinghe Town | 108 |  |
| Jilin | 7 | Jiangyuan District | 28 | Urban | Zhengcha Community | 109 | 2018/07/18-2018/08/03 |
| Jilin | 7 | Jiangyuan District | 28 | Urban | Chengqiang Subdistrict | 110 |  |
| Jilin | 7 | Jiangyuan District | 28 | Urban | Wangou Town | 111 |  |
| Jilin | 7 | Jiangyuan District | 28 | Urban | Beishan Sixth Committee | 112 |  |
| Heilongjiang | 8 | Meilisi Daur District | 29 | Urban | Gonghe Town | 113 | 2018/07/06-2018/07/16 |
| Heilongjiang | 8 | Meilisi Daur District | 29 | Urban | Meilisi Subdistrict | 114 |  |
| Heilongjiang | 8 | Meilisi Daur District | 29 | Urban | Ya'ersai Town | 115 |  |
| Heilongjiang | 8 | Meilisi Daur District | 29 | Urban | Meilisi Town | 116 |  |
| Heilongjiang | 8 | Datong District, Daqing City | 30 | Urban | Linyuan Town | 117 | 2018/06/21-2018/06/27 |
| Heilongjiang | 8 | Datong District, Daqing City | 30 | Urban | Bajinzi Township | 118 |  |
| Heilongjiang | 8 | Datong District, Daqing City | 30 | Urban | Datong Town Subdistrict | 119 |  |
| Heilongjiang | 8 | Datong District, Daqing City | 30 | Urban | Laoshantou Township | 120 |  |
| Heilongjiang | 8 | Muleng City, Mudanjiang City | 31 | Rural | Bamiantong Town | 121 | 2018/06/06-2018/06/15 |
| Heilongjiang | 8 | Muleng City, Mudanjiang City | 31 | Rural | Xingyuan Town | 122 |  |
| Heilongjiang | 8 | Muleng City, Mudanjiang City | 31 | Rural | Maqiaohe Town | 123 |  |
| Heilongjiang | 8 | Muleng City, Mudanjiang City | 31 | Rural | Xiachengzi Town | 124 |  |
| Heilongjiang | 8 | Suihua City, Zhaodong City | 32 | Rural | Wuliming Town | 125 | 2018/07/17-2018/07/24 |
| Heilongjiang | 8 | Suihua City, Zhaodong City | 32 | Rural | Songzhan Town | 126 |  |
| Heilongjiang | 8 | Suihua City, Zhaodong City | 32 | Rural | Yuejin Township | 127 |  |
| Heilongjiang | 8 | Suihua City, Zhaodong City | 32 | Rural | Sizhan Town | 128 |  |
| Shanghai | 9 | Huangpu District | 33 | Urban | Yuyuan Garden | 129 | 2019/06/19-2019/07/27 |
| Shanghai | 9 | Huangpu District | 33 | Urban | Bund streets | 130 |  |
| Shanghai | 9 | Huangpu District | 33 | Urban | Laoximen | 131 |  |
| Shanghai | 9 | Huangpu District | 33 | Urban | Wuliqiao Street | 132 |  |
| Shanghai | 9 | Putuo District | 34 | Urban | Taopu | 133 | 2019/06/15-2019/06/30 |
| Shanghai | 9 | Putuo District | 34 | Urban | Zhenru Town | 134 |  |
| Shanghai | 9 | Putuo District | 34 | Urban | Changfeng | 135 |  |
| Shanghai | 9 | Putuo District | 34 | Urban | Yichuan | 136 |  |
| Shanghai | 9 | Songjiang District | 35 | Urban | Dongjing Town | 137 | 2019/05/31-2019/08/16 |
| Shanghai | 9 | Songjiang District | 35 | Urban | Zhongshan Street | 138 |  |
| Shanghai | 9 | Songjiang District | 35 | Urban | Fangsong Street | 139 |  |
| Shanghai | 9 | Songjiang District | 35 | Urban | Chedun Town | 140 |  |
| Shanghai | 9 | Fengxian District | 36 | Urban | Haiwan Town | 141 | 2019/06/22-2019/07/14 |
| Shanghai | 9 | Fengxian District | 36 | Urban | Xidu Town | 142 |  |
| Shanghai | 9 | Fengxian District | 36 | Urban | Zhelin Town | 143 |  |
| Shanghai | 9 | Fengxian District | 36 | Urban | Fengcheng Town | 144 |  |
| Jiangsu | 10 | Xuanwu District, Nanjing City | 37 | Urban | Xinjiekou Street | 145 | 2019/09/07-2019/10/20 |
| Jiangsu | 10 | Xuanwu District, Nanjing City | 37 | Urban | Meiyuanxincun Street | 146 |  |
| Jiangsu | 10 | Xuanwu District, Nanjing City | 37 | Urban | Hongshan Street | 147 |  |
| Jiangsu | 10 | Xuanwu District, Nanjing City | 37 | Urban | Xuanwu Lake Street | 148 |  |
| Jiangsu | 10 | Jia Wang District, Xuzhou City | 38 | Urban | Dawu Town | 149 | 2019/05/07-2019/05/25 |
| Jiangsu | 10 | Jia Wang District, Xuzhou City | 38 | Urban | Biantang Town | 150 |  |
| Jiangsu | 10 | Jia Wang District, Xuzhou City | 38 | Urban | Tashan Town | 151 |  |
| Jiangsu | 10 | Jia Wang District, Xuzhou City | 38 | Urban | Xiaqiao Street | 152 |  |
| Jiangsu | 10 | Changshu City, Suzhou City | 39 | Rural | Shanghu Town | 153 | 2019/06/16-2019/07/14 |
| Jiangsu | 10 | Changshu City, Suzhou City | 39 | Rural | Shajiabang Town | 154 |  |
| Jiangsu | 10 | Changshu City, Suzhou City | 39 | Rural | Changfu Street | 155 |  |
| Jiangsu | 10 | Changshu City, Suzhou City | 39 | Rural | Qinchuan Street | 156 |  |
| Jiangsu | 10 | Hai'an City | 40 | Rural | Libao | 157 | 2019/08/13-2019/08/22 |
| Jiangsu | 10 | Hai'an City | 40 | Rural | Chengdong | 158 |  |
| Jiangsu | 10 | Hai'an City | 40 | Rural | Qutang | 159 |  |
| Jiangsu | 10 | Hai'an City | 40 | Rural | Yazhou | 160 |  |
| Zhejiang | 11 | Haining City | 41 | Rural | Haichang Street | 161 | 2018/07/05-2018/07/27 |
| Zhejiang | 11 | Haining City | 41 | Rural | Xieqiao Town | 162 |  |
| Zhejiang | 11 | Haining City | 41 | Rural | Huangwan Town | 163 |  |
| Zhejiang | 11 | Haining City | 41 | Rural | Yuanhua Town | 164 |  |
| Zhejiang | 11 | Tongxiang City | 42 | Rural | Zhouquan Town | 165 | 2018/03/16-2018/06/29 |
| Zhejiang | 11 | Tongxiang City | 42 | Rural | Wuzhen Town | 166 |  |
| Zhejiang | 11 | Tongxiang City | 42 | Rural | Gaoqiao Town | 167 |  |
| Zhejiang | 11 | Tongxiang City | 42 | Rural | Chongfu Town | 168 |  |
| Zhejiang | 11 | Keqiao District | 43 | Urban | Xialu Town | 169 | 2018/07/18-2018/08/03 |
| Zhejiang | 11 | Keqiao District | 43 | Urban | Anchang Street | 170 |  |
| Zhejiang | 11 | Keqiao District | 43 | Urban | Pingshui Town | 171 |  |
| Zhejiang | 11 | Keqiao District | 43 | Urban | Qixian Street | 172 |  |
| Zhejiang | 11 | Wucheng District | 44 | Urban | Chengzhong Street | 173 | 2018/08/09-2018/08/29 |
| Zhejiang | 11 | Wucheng District | 44 | Urban | Yafan | 174 |  |
| Zhejiang | 11 | Wucheng District | 44 | Urban | Yangbu Town | 175 |  |
| Zhejiang | 11 | Wucheng District | 44 | Urban | Qianxi Township | 176 |  |
| Anhui | 12 | Yushan District, Ma'anshan City | 45 | Urban | Yintang Town | 177 | 2018/11/06-2018/11/13 |
| Anhui | 12 | Yushan District, Ma'anshan City | 45 | Urban | Xiangshan Town | 178 |  |
| Anhui | 12 | Yushan District, Ma'anshan City | 45 | Urban | Anmin Street | 179 |  |
| Anhui | 12 | Yushan District, Ma'anshan City | 45 | Urban | Yushan Street | 180 |  |
| Anhui | 12 | Tianchang City | 46 | Rural | Yangcun Town | 181 | 2018/12/05-2018/12/12 |
| Anhui | 12 | Tianchang City | 46 | Rural | Yeshan Town | 182 |  |
| Anhui | 12 | Tianchang City | 46 | Rural | Chajian Town | 183 |  |
| Anhui | 12 | Tianchang City | 46 | Rural | Zhengji Town | 184 |  |
| Anhui | 12 | Yingzhou District | 47 | Urban | Sanhe Town | 185 | 2018/10/16-2018/10/23 |
| Anhui | 12 | Yingzhou District | 47 | Urban | Gulou Office | 186 |  |
| Anhui | 12 | Yingzhou District | 47 | Urban | Xihu Town | 187 |  |
| Anhui | 12 | Yingzhou District | 47 | Urban | Yingxi Office | 188 |  |
| Anhui | 12 | Shou County | 48 | Rural | Yankou | 189 | 2018/11/21-2018/11/29 |
| Anhui | 12 | Shou County | 48 | Rural | Yinghe | 190 |  |
| Anhui | 12 | Shou County | 48 | Rural | Xiaodian | 191 |  |
| Anhui | 12 | Shou County | 48 | Rural | Sanjiao | 192 |  |
| Fujian | 13 | Hui'an County | 49 | Rural | Luoyang Town | 193 | 2019/07/20-2019/07/28 |
| Fujian | 13 | Hui'an County | 49 | Rural | Huangtang Town | 194 |  |
| Fujian | 13 | Hui'an County | 49 | Rural | Dongqiao Town | 195 |  |
| Fujian | 13 | Hui'an County | 49 | Rural | Chongwu Town | 196 |  |
| Fujian | 13 | Xinluo District, Longyan City | 50 | Urban | Dongcheng Street | 197 | 2019/10/23-2019/11/07 |
| Fujian | 13 | Xinluo District, Longyan City | 50 | Urban | Zhongcheng Street | 198 |  |
| Fujian | 13 | Xinluo District, Longyan City | 50 | Urban | Xipi Street | 199 |  |
| Fujian | 13 | Xinluo District, Longyan City | 50 | Urban | Longmen Street | 200 |  |
| Fujian | 13 | Yongding County | 51 | Urban | Hulei Town | 201 | 2019/09/18-2019/10/22 |
| Fujian | 13 | Yongding County | 51 | Urban | Daxi Township | 202 |  |
| Fujian | 13 | Yongding County | 51 | Urban | Fengcheng Town | 203 |  |
| Fujian | 13 | Yongding County | 51 | Urban | Xiayang Town | 204 |  |
| Fujian | 13 | Pingnan County | 52 | Rural | Daixi Town | 205 | 2019/07/02-2019/07/16 |
| Fujian | 13 | Pingnan County | 52 | Rural | Lingxia Township | 206 |  |
| Fujian | 13 | Pingnan County | 52 | Rural | Shuangxi Township | 207 |  |
| Fujian | 13 | Pingnan County | 52 | Rural | Changqiao Town | 208 |  |
| Jiangxi | 14 | Donghu District, Nanchang City | 53 | Urban | Pengjiaqiao Street | 209 | 2018/09/26-2018/10/31 |
| Jiangxi | 14 | Donghu District, Nanchang City | 53 | Urban | Yuzhang Street | 210 |  |
| Jiangxi | 14 | Donghu District, Nanchang City | 53 | Urban | Tengwangge Street | 211 |  |
| Jiangxi | 14 | Donghu District, Nanchang City | 53 | Urban | Baihuazhou Street | 212 |  |
| Jiangxi | 14 | Yushui District | 54 | Urban | Chengnan | 213 | 2019/02/21-2019/02/28 |
| Jiangxi | 14 | Yushui District | 54 | Urban | Chengbei | 214 |  |
| Jiangxi | 14 | Yushui District | 54 | Urban | Luofang | 215 |  |
| Jiangxi | 14 | Yushui District | 54 | Urban | Shuibei | 216 |  |
| Jiangxi | 14 | Shanggao County | 55 | Rural | Xujiadu | 217 | 2018/11/14-2018/11/25 |
| Jiangxi | 14 | Shanggao County | 55 | Rural | Luzhou | 218 |  |
| Jiangxi | 14 | Shanggao County | 55 | Rural | Xujiadu | 219 |  |
| Jiangxi | 14 | Shanggao County | 55 | Rural | Sixi | 220 |  |
| Jiangxi | 14 | Hengfeng County | 56 | Rural | Xing'an Street | 221 | 2018/12/21-2018/12/28 |
| Jiangxi | 14 | Hengfeng County | 56 | Rural | Geyuan | 222 |  |
| Jiangxi | 14 | Hengfeng County | 56 | Rural | Longmen Township | 223 |  |
| Jiangxi | 14 | Hengfeng County | 56 | Rural | Lianhe | 224 |  |
| Shandong | 15 | Zhangqiu City | 57 | Urban | Longshan | 225 | 2018/06/14-2018/06/26 |
| Shandong | 15 | Zhangqiu City | 57 | Urban | Xianggong | 226 |  |
| Shandong | 15 | Zhangqiu City | 57 | Urban | Diao Town | 227 |  |
| Shandong | 15 | Zhangqiu City | 57 | Urban | Huanghe | 228 |  |
| Shandong | 15 | Yiyuan County | 58 | Rural | Zhangjiapo | 229 | 2018/07/31-2018/08/08 |
| Shandong | 15 | Yiyuan County | 58 | Rural | Xili Town | 230 |  |
| Shandong | 15 | Yiyuan County | 58 | Rural | Zhongzhuang | 231 |  |
| Shandong | 15 | Yiyuan County | 58 | Rural | Lucun Town | 232 |  |
| Shandong | 15 | Rushan City | 59 | Rural | Xiacun | 233 | 2018/08/21-2018/08/30 |
| Shandong | 15 | Rushan City | 59 | Rural | Baishatan | 234 |  |
| Shandong | 15 | Rushan City | 59 | Rural | Nanhuang | 235 |  |
| Shandong | 15 | Rushan City | 59 | Rural | Rushankou | 236 |  |
| Shandong | 15 | Laicheng District, Laiwu City | 60 | Urban | Niuquan Town | 237 | 2018/07/17-2018/07/26 |
| Shandong | 15 | Laicheng District, Laiwu City | 60 | Urban | Gaozhuang Subdistrict | 238 |  |
| Shandong | 15 | Laicheng District, Laiwu City | 60 | Urban | Fangxia Town | 239 |  |
| Shandong | 15 | Laicheng District, Laiwu City | 60 | Urban | Kou Town | 240 |  |
| Shandong | 15 | Gaotang County, Liaocheng City | 61 | Rural | Zhaozhaizi Town | 241 | 2018/07/03-2018/07/12 |
| Shandong | 15 | Gaotang County, Liaocheng City | 61 | Rural | Yinji Town | 242 |  |
| Shandong | 15 | Gaotang County, Liaocheng City | 61 | Rural | Qingping Town | 243 |  |
| Shandong | 15 | Gaotang County, Liaocheng City | 61 | Rural | Guhe Town | 244 |  |
| Henan | 16 | Jili District | 62 | Urban | Heyang | 245 | 2018/05/24-2018/07/02 |
| Henan | 16 | Jili District | 62 | Urban | Jili Subdistrict | 246 |  |
| Henan | 16 | Jili District | 62 | Urban | Kangle Community Subdistrict | 247 |  |
| Henan | 16 | Jili District | 62 | Urban | Xixiayuan Subdistrict | 248 |  |
| Henan | 16 | Hua County | 63 | Rural | Wangu Town | 249 | 2018/07/04-2018/07/11 |
| Henan | 16 | Hua County | 63 | Rural | Daokou Town | 250 |  |
| Henan | 16 | Hua County | 63 | Rural | Laodian town | 251 |  |
| Henan | 16 | Hua County | 63 | Rural | Dazhai Township | 252 |  |
| Henan | 16 | Weidu District | 64 | Urban | Xiguan Subdistrict | 253 | 2018/06/21-2018/06/30 |
| Henan | 16 | Weidu District | 64 | Urban | Wuyi Road Subdistrict | 254 |  |
| Henan | 16 | Weidu District | 64 | Urban | Wenfeng Subdistrict | 255 |  |
| Henan | 16 | Weidu District | 64 | Urban | Xinxing Subdistrict | 256 |  |
| Henan | 16 | Lingbao City | 65 | Rural | Chengguan Town | 257 | 2018/05/28-2018/06/07 |
| Henan | 16 | Lingbao City | 65 | Rural | Yangdian Town | 258 |  |
| Henan | 16 | Lingbao City | 65 | Rural | Yinzhuang Town | 259 |  |
| Henan | 16 | Lingbao City | 65 | Rural | Yangping Town | 260 |  |
| Henan | 16 | Tanghe County | 66 | Rural | Tonghe Township | 261 | 2018/06/12-2018/06/26 |
| Henan | 16 | Tanghe County | 66 | Rural | Cangtai Town | 262 |  |
| Henan | 16 | Tanghe County | 66 | Rural | Guotan Town | 263 |  |
| Henan | 16 | Tanghe County | 66 | Rural | Chengjiao Township | 264 |  |
| Hubei | 17 | Wujiagang District, Yichang City | 67 | Urban | Baotahe Subdistrict | 265 | 2019/07/16-2019/07/23 |
| Hubei | 17 | Wujiagang District, Yichang City | 67 | Urban | Wanshouqiao Subdistrict | 266 |  |
| Hubei | 17 | Wujiagang District, Yichang City | 67 | Urban | Wujia Township | 267 |  |
| Hubei | 17 | Wujiagang District, Yichang City | 67 | Urban | Wujiagang Subdistrict | 268 |  |
| Hubei | 17 | Xiaonan District | 68 | Urban | Shuyuan Subdistrict | 269 | 2019/07/02-2019/08/02 |
| Hubei | 17 | Xiaonan District | 68 | Urban | Xinhua Subdistrict | 270 |  |
| Hubei | 17 | Xiaonan District | 68 | Urban | Shuyuan Subdistrict | 271 |  |
| Hubei | 17 | Xiaonan District | 68 | Urban | Chezhan Subdistrict | 272 |  |
| Hubei | 17 | Yunmeng County | 69 | Rural | Qingminghe Township | 273 | 2019/06/18-2019/11/12 |
| Hubei | 17 | Yunmeng County | 69 | Rural | Geputan Town | 274 |  |
| Hubei | 17 | Yunmeng County | 69 | Rural | Chengguan Town | 275 |  |
| Hubei | 17 | Yunmeng County | 69 | Rural | Wupu Town | 276 |  |
| Hubei | 17 | Macheng City, Huanggang City | 70 | Rural | Fuzihe Town | 277 | 2019/07/30-2019/08/06 |
| Hubei | 17 | Macheng City, Huanggang City | 70 | Rural | Yantianhe Town | 278 |  |
| Hubei | 17 | Macheng City, Huanggang City | 70 | Rural | Muzidian Town | 279 |  |
| Hubei | 17 | Macheng City, Huanggang City | 70 | Rural | Shunhe Town | 280 |  |
| Hunan | 18 | Tianxin District | 71 | Urban | Xianfeng Subdistrict | 281 | 2018/10/17-2018/11/10 |
| Hunan | 18 | Tianxin District | 71 | Urban | Pozi Street Subdistrict | 282 |  |
| Hunan | 18 | Tianxin District | 71 | Urban | Heishipu Subdistrict | 283 |  |
| Hunan | 18 | Tianxin District | 71 | Urban | Yunan Street Subdistrict | 284 |  |
| Hunan | 18 | Lusong District, Zhuzhou City | 72 | Urban | Hejiatu Subdistrict | 285 | 2018/12/13-2018/12/24 |
| Hunan | 18 | Lusong District, Zhuzhou City | 72 | Urban | Longquan Subdistrict | 286 |  |
| Hunan | 18 | Lusong District, Zhuzhou City | 72 | Urban | Qingyun | 287 |  |
| Hunan | 18 | Lusong District, Zhuzhou City | 72 | Urban | Dongjiaduan | 288 |  |
| Hunan | 18 | Xiangtan County | 73 | Rural | Tanjiashan Town | 289 | 2018/11/26-2018/12/07 |
| Hunan | 18 | Xiangtan County | 73 | Rural | Yangjiaqiao Town | 290 |  |
| Hunan | 18 | Xiangtan County | 73 | Rural | Meilinqiao Town | 291 |  |
| Hunan | 18 | Xiangtan County | 73 | Rural | Wushi Town | 292 |  |
| Hunan | 18 | Shaodong County, Shaoyang City | 74 | Rural | Lianqiao | 293 | 2019/02/26-2019/03/09 |
| Hunan | 18 | Shaodong County, Shaoyang City | 74 | Rural | Liuguangling Town | 294 |  |
| Hunan | 18 | Shaodong County, Shaoyang City | 74 | Rural | Xianchaqiao | 295 |  |
| Hunan | 18 | Shaodong County, Shaoyang City | 74 | Rural | Shuangfeng | 296 |  |
| Guangdong | 19 | Yuexiu District, Guangzhou City | 75 | Urban | Dongshan Subdistrict | 297 | 2018/06/19-2018/07/04 |
| Guangdong | 19 | Yuexiu District, Guangzhou City | 75 | Urban | Huanghuagang Subdistrict | 298 |  |
| Guangdong | 19 | Yuexiu District, Guangzhou City | 75 | Urban | Hongqiao Subdistrict | 299 |  |
| Guangdong | 19 | Yuexiu District, Guangzhou City | 75 | Urban | People's Street Subdistrict | 300 |  |
| Guangdong | 19 | Sihui City | 76 | Rural | Shigou Town | 301 | 2018/09/12-2018/09/25 |
| Guangdong | 19 | Sihui City | 76 | Rural | Chengzhong Subdistrict | 302 |  |
| Guangdong | 19 | Sihui City | 76 | Rural | Jianggu Town | 303 |  |
| Guangdong | 19 | Sihui City | 76 | Rural | Dongcheng Subdistrict | 304 |  |
| Guangdong | 19 | Wuhua County | 77 | Rural | Shuizhai Town | 305 | 2018/08/01-2018/08/10 |
| Guangdong | 19 | Wuhua County | 77 | Rural | Changbu Town | 306 |  |
| Guangdong | 19 | Wuhua County | 77 | Rural | Zhuanshui Town | 307 |  |
| Guangdong | 19 | Wuhua County | 77 | Rural | Mianyang Town | 308 |  |
| Guangdong | 19 | Cheng District | 78 | Urban | Jiesheng Town | 309 | 2018/07/17-2018/07/26 |
| Guangdong | 19 | Cheng District | 78 | Urban | Xiangzhou Subdistrict | 310 |  |
| Guangdong | 19 | Cheng District | 78 | Urban | Fengshan Subdistrict | 311 |  |
| Guangdong | 19 | Cheng District | 78 | Urban | Xingang Subdistrict | 312 |  |
| Guangxi | 20 | Xingning District | 79 | Urban | Santang Town | 313 | 2019/04/26-2019/05/15 |
| Guangxi | 20 | Xingning District | 79 | Urban | Minsheng Subdistrict | 314 |  |
| Guangxi | 20 | Xingning District | 79 | Urban | Chaoyang Subdistrict | 315 |  |
| Guangxi | 20 | Xingning District | 79 | Urban | Xingdong Subdistrict | 316 |  |
| Guangxi | 20 | Binyang County | 80 | Rural | Xinqiao Town | 317 | 2019/06/22-2019/06/29 |
| Guangxi | 20 | Binyang County | 80 | Rural | Gula Town | 318 |  |
| Guangxi | 20 | Binyang County | 80 | Rural | Binzhou Town | 319 |  |
| Guangxi | 20 | Binyang County | 80 | Rural | Daqiao Town | 320 |  |
| Guangxi | 20 | Qinbei District, Qinzhou City | 81 | Urban | Zicai Subdistrict | 321 | 2019/06/12-2019/06/19 |
| Guangxi | 20 | Qinbei District, Qinzhou City | 81 | Urban | Pingji Town | 322 |  |
| Guangxi | 20 | Qinbei District, Qinzhou City | 81 | Urban | Xiaodong Town | 323 |  |
| Guangxi | 20 | Qinbei District, Qinzhou City | 81 | Urban | Nameng Town | 324 |  |
| Guangxi | 20 | Lingyun County | 82 | Rural | Luolou Town | 325 | 2019/05/28-2019/06/05 |
| Guangxi | 20 | Lingyun County | 82 | Rural | Lingzhan Township | 326 |  |
| Guangxi | 20 | Lingyun County | 82 | Rural | Xiajia Town | 327 |  |
| Guangxi | 20 | Lingyun County | 82 | Rural | Sicheng Town | 328 |  |
| Hainan | 21 | Meilan District | 83 | Urban | People's Street Subdistrict | 329 | 2018/10/08-2019/03/30 |
| Hainan | 21 | Meilan District | 83 | Urban | Yanfeng Town | 330 |  |
| Hainan | 21 | Meilan District | 83 | Urban | Haidian Subdistrict | 331 |  |
| Hainan | 21 | Meilan District | 83 | Urban | Lantian Subdistrict | 332 |  |
| Hainan | 21 | Sanya City | 84 | Urban | Haitang Bay | 333 | 2018/09/28-2018/11/15 |
| Hainan | 21 | Sanya City | 84 | Urban | Hedong District | 334 |  |
| Hainan | 21 | Sanya City | 84 | Urban | Hexi | 335 |  |
| Hainan | 21 | Sanya City | 84 | Urban | Huangyuan Village | 336 |  |
| Hainan | 21 | Wanning City | 85 | Rural | Damao Town | 337 | 2018/12/11-2019/01/10 |
| Hainan | 21 | Wanning City | 85 | Rural | Nanqiao Town | 338 |  |
| Hainan | 21 | Wanning City | 85 | Rural | Hou'an Town | 339 |  |
| Hainan | 21 | Wanning City | 85 | Rural | Shangen Town | 340 |  |
| Hainan | 21 | Ding'an County | 86 | Rural | State-owned Nanhai Farm | 341 | 2018/12/12-2019/04/08 |
| Hainan | 21 | Ding'an County | 86 | Rural | Huangzhu Town | 342 |  |
| Hainan | 21 | Ding'an County | 86 | Rural | Longhe Town | 343 |  |
| Hainan | 21 | Ding'an County | 86 | Rural | Fuwen | 344 |  |
| Chongqing | 22 | Yuzhong District | 87 | Urban | Jiefangbei | 345 | 2018/07/30-2018/08/14 |
| Chongqing | 22 | Yuzhong District | 87 | Urban | Daxigou | 346 |  |
| Chongqing | 22 | Yuzhong District | 87 | Urban | Shangqingsi | 347 |  |
| Chongqing | 22 | Yuzhong District | 87 | Urban | Lianglukou | 348 |  |
| Chongqing | 22 | Qijiang District | 88 | Urban | Wenlong | 349 | 2018/08/28-2018/09/07 |
| Chongqing | 22 | Qijiang District | 88 | Urban | Sanjiang | 350 |  |
| Chongqing | 22 | Qijiang District | 88 | Urban | Shijiao Town | 351 |  |
| Chongqing | 22 | Qijiang District | 88 | Urban | Zhuantang Town | 352 |  |
| Chongqing | 22 | Dazu County | 89 | Urban | Longgang | 353 | 2018/07/17-2018/08/07 |
| Chongqing | 22 | Dazu County | 89 | Urban | Baoding Town | 354 |  |
| Chongqing | 22 | Dazu County | 89 | Urban | Sanqu Town | 355 |  |
| Chongqing | 22 | Dazu County | 89 | Urban | Longshui Town | 356 |  |
| Chongqing | 22 | Jiangjin District | 90 | Urban | Jijiang Subdistrict | 357 | 2018/08/13-2018/08/27 |
| Chongqing | 22 | Jiangjin District | 90 | Urban | Youxi Town | 358 |  |
| Chongqing | 22 | Jiangjin District | 90 | Urban | Shimen Town | 359 |  |
| Chongqing | 22 | Jiangjin District | 90 | Urban | Caijia Town | 360 |  |
| Sichuan | 23 | Qingyang District, Chengdu City | 91 | Urban | Funan Subdistrict | 361 | 2019/05/18-2019/05/25 |
| Sichuan | 23 | Qingyang District, Chengdu City | 91 | Urban | Xinhua West Road | 362 |  |
| Sichuan | 23 | Qingyang District, Chengdu City | 91 | Urban | Xiyuhe Subdistrict | 363 |  |
| Sichuan | 23 | Qingyang District, Chengdu City | 91 | Urban | Huangtianba Subdistrict | 364 |  |
| Sichuan | 23 | Pengzhou City | 92 | Rural | Aoping Town | 365 | 2019/05/28-2019/06/06 |
| Sichuan | 23 | Pengzhou City | 92 | Rural | Sanjie Town | 366 |  |
| Sichuan | 23 | Pengzhou City | 92 | Rural | Lichun Town | 367 |  |
| Sichuan | 23 | Pengzhou City | 92 | Rural | Tianpeng Town | 368 |  |
| Sichuan | 23 | Lizhou District, Guangyuan City | 93 | Urban | Dongba Subdistrict | 369 | 2019/05/09-2019/05/22 |
| Sichuan | 23 | Lizhou District, Guangyuan City | 93 | Urban | Rongshan Town | 370 |  |
| Sichuan | 23 | Lizhou District, Guangyuan City | 93 | Urban | Dashi Town | 371 |  |
| Sichuan | 23 | Lizhou District, Guangyuan City | 93 | Urban | Baolun Town | 372 |  |
| Sichuan | 23 | Zizhong County | 94 | Rural | Sujiawan Town | 373 | 2019/04/24-2019/05/07 |
| Sichuan | 23 | Zizhong County | 94 | Rural | Gongmin Town | 374 |  |
| Sichuan | 23 | Zizhong County | 94 | Rural | Guide Town | 375 |  |
| Sichuan | 23 | Zizhong County | 94 | Rural | Yinshan Town | 376 |  |
| Sichuan | 23 | Yibin County, Yibin City | 95 | Rural | Baixi Town | 377 | 2019/04/16-2019/04/23 |
| Sichuan | 23 | Yibin County, Yibin City | 95 | Rural | Guanyin Town | 378 |  |
| Sichuan | 23 | Yibin County, Yibin City | 95 | Rural | Juexi Town | 379 |  |
| Sichuan | 23 | Yibin County, Yibin City | 95 | Rural | Nixi Town | 380 |  |
| Guizhou | 24 | Liuzhi Special District, Liupanshui City | 96 | Urban | Langdai Town | 381 | 2019/12/10-2019/12/17 |
| Guizhou | 24 | Liuzhi Special District, Liupanshui City | 96 | Urban | Jingkou Township | 382 |  |
| Guizhou | 24 | Liuzhi Special District, Liupanshui City | 96 | Urban | Yanjiao Town | 383 |  |
| Guizhou | 24 | Liuzhi Special District, Liupanshui City | 96 | Urban | Zhexi Township | 384 |  |
| Guizhou | 24 | Honghuagang District, Zunyi City | 97 | Urban | Laocheng Subdistrict | 385 | 2019/12/10-2019/12/23 |
| Guizhou | 24 | Honghuagang District, Zunyi City | 97 | Urban | Zhoushuiqiao | 386 |  |
| Guizhou | 24 | Honghuagang District, Zunyi City | 97 | Urban | Beijing Road Community | 387 |  |
| Guizhou | 24 | Honghuagang District, Zunyi City | 97 | Urban | Zhongzhuang Town | 388 |  |
| Guizhou | 24 | Meitan County | 98 | Rural | Yuquan Subdistrict | 389 | 2019/11/18-2019/11/28 |
| Guizhou | 24 | Meitan County | 98 | Rural | Meijiang Subdistrict | 390 |  |
| Guizhou | 24 | Meitan County | 98 | Rural | Gaotai Town | 391 |  |
| Guizhou | 24 | Meitan County | 98 | Rural | Xinglong Town | 392 |  |
| Guizhou | 24 | Qixingguan District | 99 | Urban | Xiaojichang Town | 393 | 2019/12/11-2019/12/26 |
| Guizhou | 24 | Qixingguan District | 99 | Urban | Changchun Town | 394 |  |
| Guizhou | 24 | Qixingguan District | 99 | Urban | Tianba Town | 395 |  |
| Guizhou | 24 | Qixingguan District | 99 | Urban | Duipo Town | 396 |  |
| Yunnan | 25 | Chuxiong City | 100 | Rural | Mishi Street Community Health Service Center | 397 | 2019/09/19-2019/09/27 |
| Yunnan | 25 | Chuxiong City | 100 | Rural | Beipu Community Health Service Center | 398 |  |
| Yunnan | 25 | Chuxiong City | 100 | Rural | Donghua Town | 399 |  |
| Yunnan | 25 | Chuxiong City | 100 | Rural | Zhongshan Town | 400 |  |
| Yunnan | 25 | Gejiu city | 101 | Rural | Chengqu Subdistrict | 401 | 2019/10/16-2019/10/23 |
| Yunnan | 25 | Gejiu city | 101 | Rural | Jijie Town | 402 |  |
| Yunnan | 25 | Gejiu city | 101 | Rural | Datun Town | 403 |  |
| Yunnan | 25 | Gejiu city | 101 | Rural | Shadian Town | 404 |  |
| Yunnan | 25 | Xiangyun County | 102 | Rural | Xiazhuang Town | 405 | 2019/08/27-2019/09/02 |
| Yunnan | 25 | Xiangyun County | 102 | Rural | Yunnanyi Town | 406 |  |
| Yunnan | 25 | Xiangyun County | 102 | Rural | Liuchang Town | 407 |  |
| Yunnan | 25 | Xiangyun County | 102 | Rural | Xiangcheng Town | 408 |  |
| Yunnan | 25 | Lanping County, Nujiang Prefecture | 103 | Rural | Lajing Town | 409 | 2019/10/29-2019/11/27 |
| Yunnan | 25 | Lanping County, Nujiang Prefecture | 103 | Rural | Yingpan Town | 410 |  |
| Yunnan | 25 | Lanping County, Nujiang Prefecture | 103 | Rural | Tongdian Town | 411 |  |
| Yunnan | 25 | Lanping County, Nujiang Prefecture | 103 | Rural | Hexi Township | 412 |  |
| Tibet | 26 | Chengguan District | 104 | Urban | Baguo Subdistrict | 413 | 2019/05/18-2019/05/30 |
| Tibet | 26 | Chengguan District | 104 | Urban | Gamagongsang | 414 |  |
| Tibet | 26 | Chengguan District | 104 | Urban | Gongdelin Subdistrict | 415 |  |
| Tibet | 26 | Chengguan District | 104 | Urban | Zhaxi Subdistrict | 416 |  |
| Tibet | 26 | Bayi District | 105 | Urban | Bayi Town | 417 | 2019/07/06-2019/07/10 |
| Tibet | 26 | Bayi District | 105 | Urban | Linzhi Town | 418 |  |
| Tibet | 26 | Bayi District | 105 | Urban | Bujiu Township | 419 |  |
| Tibet | 26 | Bayi District | 105 | Urban | Mirui Township | 420 |  |
| Tibet | 26 | Gongbujiangda County | 106 | Rural | Gongbujiangda Town | 421 | 2019/07/15-2019/07/17 |
| Tibet | 26 | Gongbujiangda County | 106 | Rural | Jiangda Township | 422 |  |
| Tibet | 26 | Gongbujiangda County | 106 | Rural | Jinda Town | 423 |  |
| Tibet | 26 | Gongbujiangda County | 106 | Rural | Bahe Town | 424 |  |
| Shaanxi | 27 | Lianhu District, Xi'an City | 107 | Urban | Beiyuanmen Subdistrict | 425 | 2019/04/11-2019/04/28 |
| Shaanxi | 27 | Lianhu District, Xi'an City | 107 | Urban | Huanxi Subdistrict | 426 |  |
| Shaanxi | 27 | Lianhu District, Xi'an City | 107 | Urban | Youth Road Subdistrict | 427 |  |
| Shaanxi | 27 | Lianhu District, Xi'an City | 107 | Urban | Taoyuan Subdistrict | 428 |  |
| Shaanxi | 27 | Wangyi District, Tongchuan City | 108 | Urban | Hongqi Subdistrict | 429 | 2019/05/06-2019/05/14 |
| Shaanxi | 27 | Wangyi District, Tongchuan City | 108 | Urban | Qiyi Subdistrict | 430 |  |
| Shaanxi | 27 | Wangyi District, Tongchuan City | 108 | Urban | Youth Road Subdistrict | 431 |  |
| Shaanxi | 27 | Wangyi District, Tongchuan City | 108 | Urban | Huangbao Town | 432 |  |
| Shaanxi | 27 | Mei County | 109 | Rural | Hengqu Town | 433 | 2019/05/23-2019/05/30 |
| Shaanxi | 27 | Mei County | 109 | Rural | Yingtou Town | 434 |  |
| Shaanxi | 27 | Mei County | 109 | Rural | Shoushan Subdistrict | 435 |  |
| Shaanxi | 27 | Mei County | 109 | Rural | Tangyu Town | 436 |  |
| Shaanxi | 27 | Jingyang County | 110 | Rural | Zhongzhang Town | 437 | 2019/05/15-2019/05/22 |
| Shaanxi | 27 | Jingyang County | 110 | Rural | Qiaodi Town | 438 |  |
| Shaanxi | 27 | Jingyang County | 110 | Rural | Xinglong Town | 439 |  |
| Shaanxi | 27 | Jingyang County | 110 | Rural | Kou Town | 440 |  |
| Gansu | 28 | Jingtai County, Baiyin City | 111 | Rural | Manshuitan Township | 441 | 2018/07/02-2018/07/24 |
| Gansu | 28 | Jingtai County, Baiyin City | 111 | Rural | Wufo Township | 442 |  |
| Gansu | 28 | Jingtai County, Baiyin City | 111 | Rural | Tiaoshan Town | 443 |  |
| Gansu | 28 | Jingtai County, Baiyin City | 111 | Rural | Yitiaoshan Town | 444 |  |
| Gansu | 28 | Liangzhou District, Wuwei City | 112 | Urban | Daliu Town | 445 | 2018/07/19-2018/11/28 |
| Gansu | 28 | Liangzhou District, Wuwei City | 112 | Urban | Xiehe Town | 446 |  |
| Gansu | 28 | Liangzhou District, Wuwei City | 112 | Urban | Jinyang Town | 447 |  |
| Gansu | 28 | Liangzhou District, Wuwei City | 112 | Urban | Fafang Town | 448 |  |
| Gansu | 28 | Ganzhou District | 113 | Urban | Wujiang Town | 449 | 2018/07/31-2018/08/10 |
| Gansu | 28 | Ganzhou District | 113 | Urban | Shangqin Town | 450 |  |
| Gansu | 28 | Ganzhou District | 113 | Urban | Mingyong Town | 451 |  |
| Gansu | 28 | Ganzhou District | 113 | Urban | Jiantan Town | 452 |  |
| Gansu | 28 | Longxi County, Dingxi City | 114 | Rural | Ronghe Community | 453 | 2018/08/19-2018/08/23 |
| Gansu | 28 | Longxi County, Dingxi City | 114 | Rural | Wenfeng Town Tobacco Company | 454 |  |
| Gansu | 28 | Longxi County, Dingxi City | 114 | Rural | Railway Grand Public Housing | 455 |  |
| Gansu | 28 | Longxi County, Dingxi City | 114 | Rural | Jinhaimingyuan | 456 |  |
| Qinghai | 29 | Chengbei District | 115 | Urban | Xiaoqiao Subdistrict | 457 | 2018/06/04-2018/07/18 |
| Qinghai | 29 | Chengbei District | 115 | Urban | Chaoyang Subdistrict | 458 |  |
| Qinghai | 29 | Chengbei District | 115 | Urban | Mafang Subdistrict | 459 |  |
| Qinghai | 29 | Chengbei District | 115 | Urban | Ershilipu Town | 460 |  |
| Qinghai | 29 | Menyuan County | 116 | Rural | Beishan Township | 461 | 2018/06/26-2018/07/05 |
| Qinghai | 29 | Menyuan County | 116 | Rural | Dongchuan Town | 462 |  |
| Qinghai | 29 | Menyuan County | 116 | Rural | Zhugu Township | 463 |  |
| Qinghai | 29 | Menyuan County | 116 | Rural | Quankou Town | 464 |  |
| Ningxia | 30 | Xingqing District, Yinchuan City | 117 | Urban | Zhangzheng Town | 465 | 2019/01/15-2019/01/30 |
| Ningxia | 30 | Xingqing District, Yinchuan City | 117 | Urban | Funing Subdistrict | 466 |  |
| Ningxia | 30 | Xingqing District, Yinchuan City | 117 | Urban | Daxin Town | 467 |  |
| Ningxia | 30 | Xingqing District, Yinchuan City | 117 | Urban | Lijing Street Subdistrict | 468 |  |
| Ningxia | 30 | Pingluo County | 118 | Rural | Chengguan Town | 469 | 2018/12/07-2018/12/23 |
| Ningxia | 30 | Pingluo County | 118 | Rural | Yaofu Town | 470 |  |
| Ningxia | 30 | Pingluo County | 118 | Rural | Tongfu Township | 471 |  |
| Ningxia | 30 | Pingluo County | 118 | Rural | Gaozhuang Township | 472 |  |
| Ningxia | 30 | Qingtongxia City, Wuzhong City | 119 | Rural | Daba Town | 473 | 2018/12/20-2019/01/04 |
| Ningxia | 30 | Qingtongxia City, Wuzhong City | 119 | Rural | Chenyuantan | 474 |  |
| Ningxia | 30 | Qingtongxia City, Wuzhong City | 119 | Rural | Qujing Town | 475 |  |
| Ningxia | 30 | Qingtongxia City, Wuzhong City | 119 | Rural | Yumin Subdistrict | 476 |  |
| Ningxia | 30 | Shapotou District | 120 | Urban | Xuanhe Town | 477 | 2019/01/09-2019/01/12 |
| Ningxia | 30 | Shapotou District | 120 | Urban | Zhenluo Town | 478 |  |
| Ningxia | 30 | Shapotou District | 120 | Urban | Binhe Town | 479 |  |
| Ningxia | 30 | Shapotou District | 120 | Urban | Yongkang Town | 480 |  |
| Xinjiang | 31 | Toksu County, Aksu Prefecture | 121 | Rural | Yiqiairike Town | 481 | 2019/12/10-2019/12/14 |
| Xinjiang | 31 | Toksu County, Aksu Prefecture | 121 | Rural | Paixianbaibazha Township | 482 |  |
| Xinjiang | 31 | Toksu County, Aksu Prefecture | 121 | Rural | Yuqikate Township | 483 |  |
| Xinjiang | 31 | Toksu County, Aksu Prefecture | 121 | Rural | Youludusibage Town | 484 |  |
| Xinjiang | 31 | Awati County, Aksu Prefecture | 122 | Rural | Baishiairike Town | 485 | 2019/12/18-2019/12/21 |
| Xinjiang | 31 | Awati County, Aksu Prefecture | 122 | Rural | Wuluquele Town | 486 |  |
| Xinjiang | 31 | Awati County, Aksu Prefecture | 122 | Rural | Yingairike Town | 487 |  |
| Xinjiang | 31 | Awati County, Aksu Prefecture | 122 | Rural | Tamutuogelake Town Health Center | 488 |  |

# **2.3 Supplementary Table 5: The sex-, age-, and rural/urban structure of Chinese adults with diabetes aged 18-74 in China in 2018-2019.**

|  | **Urban** | | **Rural** | | **Total** |
| --- | --- | --- | --- | --- | --- |
|  | **Male** | **Female** | **Male** | **Female** |  |
| **Age group, y** |  |  |  |  |  |
| 18-29 | 2887400 (2.4) | 2102121 (1.8) | 3631717 (3.0) | 3291845 (2.7) | 11913083 (9.9) |
| 30-39 | 4078360 (3.4) | 2017084 (1.7) | 3366346 (2.8) | 2387746 (2.0) | 11849536 (9.9) |
| 40-49 | 7477212 (6.2) | 3837499 (3.2) | 9680794 (8.1) | 6094459 (5.1) | 27089964 (22.6) |
| 50-59 | 8209256 (6.9) | 5704905 (4.8) | 8803757 (7.4) | 8115951 (6.8) | 30833869 (25.7) |
| 60-69 | 6015643 (5.0) | 5748827 (4.8) | 7325415 (6.1) | 8411607 (7.0) | 27501492 (23.0) |
| 70-74 | 2278661 (1.9) | 2567857 (2.1) | 2627187 (2.2) | 3087544 (2.6) | 10561249 (8.8) |
| **Total** | 30946532 (25.8) | 21978293 (18.4) | 35435216 (29.6) | 31389152 (26.2) | 119749193 (100.0) |

Data were presented as number (percentage). The structure was calculated using diabetes prevalence data obtained from the 2018-2019 cycle of the China Chronic Disease and Risk Factors Surveillance, and the population size in corresponding age, sex, and urban/rural strata of the general Chinese population in 2018.

# **3 Supplementary Table for the Study Group**

# **3.1 Supplementary Table 6: China National Diabetic Chronic Complications (DiaChronic) Study Group**

|  | **Affiliations** |
| --- | --- |
| Xiaohui Guo | Department of Endocrinology, Peking University First Hospital, Beijing, China |
| Kai Wu | Department of Endocrinology, Peking University First Hospital, Beijing, China |
| Liming Chen | Tianjin Metabolic Diseases Hospital & Tianjin Institute of Endocrinology, Tianjin Medical University, Tianjin, China |
| Demin Liu | Tianjin Metabolic Diseases Hospital & Tianjin Institute of Endocrinology, Tianjin Medical University, Tianjin, China |
| Guangyao Song | Department of Endocrinology, Hebei General Hospital, Shijiazhuang, Hebei, China |
| Linyi Shu | Department of Endocrinology, Hebei General Hospital, Shijiazhuang, Hebei, China |
| Jing Yang | Department of Endocrinology, The First Hospital of Shanxi Medical University, Taiyuan, Shanxi, China |
| Yan Wang | Department of Endocrinology, The First Hospital of Shanxi Medical University, Taiyuan, Shanxi, China |
| Dongmei Li | Department of Endocrinology, Inner Mongolia People's Hospital, Hohhot, Inner Mongolia, China |
| Qiansha Guo | Department of Endocrinology, Inner Mongolia People's Hospital, Hohhot, Inner Mongolia, China |
| Ling Li | Department of Endocrinology, Shengjing Hospital, China Medical University, Shenyang, Liaoning, China |
| Na Wu | Department of Endocrinology, Shengjing Hospital, China Medical University, Shenyang, Liaoning, China |
| Yadong Sun | Department of Endocrinology, Jilin Province People's Hospital, Changchun, Jilin, China |
| Huifang Cheng | Department of Endocrinology, Jilin Province People's Hospital, Changchun, Jilin, China |
| Hongyu Kuang | Department of Endocrinology, The First Affiliated Hospital of Harbin Medical University, Harbin, Heilongjiang, China |
| Huijuan Zhang | Department of Endocrinology, The First Affiliated Hospital of Harbin Medical University, Harbin, Heilongjiang, China |
| Weiping Jia | Department of Endocrinology and Metabolism, Shanghai Sixth People's Hospital Affiliated to Shanghai Jiao Tong University School of Medicine, Shanghai Diabetes Institute, Shanghai Key Laboratory of Diabetes Mellitus, Shanghai Clinical Center for Diabetes, Shanghai Key Clinical Center for Metabolic Disease, Shanghai, China |
| Xuhong Hou | Department of Endocrinology and Metabolism, Shanghai Sixth People's Hospital Affiliated to Shanghai Jiao Tong University School of Medicine, Shanghai Diabetes Institute, Shanghai Key Laboratory of Diabetes Mellitus, Shanghai Clinical Center for Diabetes, Shanghai Key Clinical Center for Metabolic Disease, Shanghai, China |
| Dalong Zhu | Department of Endocrinology, Drum Tower Hospital Affiliated to Nanjing University Medical School, Nanjing, Jiangsu, China |
| Jian Zhu | Department of Endocrinology, Drum Tower Hospital Affiliated to Nanjing University Medical School, Nanjing, Jiangsu, China |
| Hong Li | Department of Endocrinology, Sir Run Run Shaw Hospital Affiliated to School of Medicine, Zhejiang University, Hangzhou, Zhejiang, China |
| Fenping Zheng | Department of Endocrinology, Sir Run Run Shaw Hospital Affiliated to School of Medicine, Zhejiang University, Hangzhou, Zhejiang, China |
| Qiu Zhang | Department of Endocrinology, First Affiliated Hospital of Anhui Medical University, Hefei, Anhui, China |
| Honglin Hu | Department of Endocrinology, First Affiliated Hospital of Anhui Medical University, Hefei, Anhui, China |
| Gang Chen | Department of Endocrinology, Fujian Provincial Hospital, Fuzhou, Fujian, China |
| Xingquan Yang | Department of Endocrinology, Fujian Provincial Hospital, Fuzhou, Fujian, China |
| Xiaoyang Lai | Department of Endocrinology, The Second Affiliated Hospital of Nanchang University, Nanchang, Jiangxi, China |
| Jianping Liu | Department of Endocrinology, The Second Affiliated Hospital of Nanchang University, Nanchang, Jiangxi, China |
| Li Chen | Department of Endocrinology, Qilu Hospital of Shandong University, Jinan, Shandong, China |
| Ming Dong | Department of Endocrinology, Qilu Hospital of Shandong University, Jinan, Shandong, China |
| Zhigang Zhao | Department of Endocrinology, Yihe Hospital of Zhengzhou, Zhengzhou, Henan, China |
| Jian Liu | Department of Endocrinology, Yihe Hospital of Zhengzhou, Zhengzhou, Henan, China |
| Guangda Xiang | Department of Endocrinology, General Hospital of Central Theater Command, Wuhan, Hubei, China |
| Junxia Zhang | Department of Endocrinology, General Hospital of Central Theater Command, Wuhan, Hubei, China |
| Zhiguang Zhou | Institute of Metabolism and Endocrinology, Key Laboratory of Diabetes Immunology, Ministry of Education, National Clinical Research Center for Metabolic Diseases, The Second Xiangya Hospital and the Diabetes Center, Central South University, Changsha, Hunan, China |
| Jian Peng | Institute of Metabolism and Endocrinology, Key Laboratory of Diabetes Immunology, Ministry of Education, National Clinical Research Center for Metabolic Diseases, The Second Xiangya Hospital and the Diabetes Center, Central South University, Changsha, Hunan, China |
| Jianping Weng | Department of Endocrinology and Metabolic Disease, The Third Affiliated Hospital of Sun Yat-sen University, Guangzhou, Guangdong, China |
| Longyi Zeng | Department of Endocrinology and Metabolic Disease, The Third Affiliated Hospital of Sun Yat-sen University, Guangzhou, Guangdong, China |
| Yuzhen Liang | Department of Endocrinology, The Second Affiliated Hospital of Guangxi Medical University, Nanning, Guangxi, China |
| Guoqiao Li | Department of Endocrinology, The Second Affiliated Hospital of Guangxi Medical University, Nanning, Guangxi, China |
| Kaining Chen | Department of Endocrinology, Hainan General Hospital, Haikou, Hainan, China |
| Leweihua Lin | Department of Endocrinology, Hainan General Hospital, Haikou, Hainan, China |
| Qifu Li | Department of Endocrinology, The First Affiliated Hospital of Chongqing Medical University, Chongqing, China |
| Qingfeng Cheng | Department of Endocrinology, The First Affiliated Hospital of Chongqing Medical University, Chongqing, China |
| Xingwu Ran | Department of Endocrinology and Metabolism, West China Hospital, Sichuan University, Chengdu, Sichuan, China |
| Dawei Chen | Department of Endocrinology and Metabolism, West China Hospital, Sichuan University, Chengdu, Sichuan, China |
| Hong Li | Department of Endocrinology, First Affiliated Hospital of Kunming Medical University, Kunming, Yunnan, China |
| Xin Nian | Department of Endocrinology, First Affiliated Hospital of Kunming Medical University, Kunming, Yunnan, China |
| Lihui Yang | Department of Endocrinology, People's Hospital of Tibet Autonomous Region, Lhasa, Tibet, China |
| Shuyou Meng | Department of Endocrinology, People's Hospital of Tibet Autonomous Region, Lhasa, Tibet, China |
| Jing Xu | Department of Endocrinology, The Second Affiliated Hospital of Xi'an Jiaotong University, Xi'an, Shaanxi, China |
| Junhong Long | Department of Endocrinology, The Second Affiliated Hospital of Xi'an Jiaotong University, Xi'an, Shaanxi, China |
| Jing Liu | Department of Endocrinology, Gansu Provincial Hospital, Lanzhou, Gansu, China |
| Qi Zhang | Department of Endocrinology, Gansu Provincial Hospital, Lanzhou, Gansu, China |
| Qingxiang Dai | Geriatrics Department, Qinghai university Affiliated Hospital, Xining, Qinghai, China |
| Xiaomin Xie | Department of Endocrinology, The First People's Hospital of Yinchuan, Yinchuan, Ningxia, China |
| Guirong Bai | Department of Endocrinology, The First People's Hospital of Yinchuan, Yinchuan, Ningxia, China |
| Jun Li | Department of Endocrinology, The First Affiliated Hospital, School of medicine, Shihezi University, Shihezi, Xinjiang, China |
| Tao Li | Department of Endocrinology, The First Affiliated Hospital, School of medicine, Shihezi University, Shihezi, Xinjiang, China |
| Zhong Dong | Beijing Provincial Center for Disease Control and Prevention, Beijing, China |
| Tao Zhang | Beijing Provincial Center for Disease Control and Prevention, Beijing, China |
| Sijia Liu | Beijing Provincial Center for Disease Control and Prevention, Beijing, China |
| Yong Yang | Beijing Provincial Center for Disease Control and Prevention, Beijing, China |
| Kun Geng | Beijing Provincial Center for Disease Control and Prevention, Beijing, China |
| Wenlong Zheng | Tianjin Provincial Center for Disease Control and Prevention, Tianjin, China |
| Zhihong Li | Tianjin Provincial Center for Disease Control and Prevention, Tianjin, China |
| Hui Wang | Tianjin Provincial Center for Disease Control and Prevention, Tianjin, China |
| Zuojun Wu | Tianjin Provincial Center for Disease Control and Prevention, Tianjin, China |
| Miao Wang | Tianjin Provincial Center for Disease Control and Prevention, Tianjin, China |
| Jixin Sun | Hebei Provincial Center for Disease Control and Prevention, Hebei, China |
| Jianwei Zhou | Hebei Provincial Center for Disease Control and Prevention, Hebei, China |
| Jun An | Hebei Provincial Center for Disease Control and Prevention, Hebei, China |
| Huaqing Shen | Hebei Provincial Center for Disease Control and Prevention, Hebei, China |
| Yanfang Li | Hebei Provincial Center for Disease Control and Prevention, Hebei, China |
| Zeping Ren | Shanxi Provincial Center for Disease Control and Prevention, Shanxi, China |
| Xiuli Xue | Shanxi Provincial Center for Disease Control and Prevention, Shanxi, China |
| Shaohui Jia | Shanxi Provincial Center for Disease Control and Prevention, Shanxi, China |
| Yongping Zheng | Shanxi Provincial Center for Disease Control and Prevention, Shanxi, China |
| Jiaoxia Li | Shanxi Provincial Center for Disease Control and Prevention, Shanxi, China |
| Yonggang Qian | Inner Mongolia Provincial Center for Disease Control and Prevention, Inner Mongolia, China |
| Ping Ma | Inner Mongolia Provincial Center for Disease Control and Prevention, Inner Mongolia, China |
| Yan Su | Inner Mongolia Provincial Center for Disease Control and Prevention, Inner Mongolia, China |
| Liang Cao | Inner Mongolia Provincial Center for Disease Control and Prevention, Inner Mongolia, China |
| Xuyi Yang | Inner Mongolia Provincial Center for Disease Control and Prevention, Inner Mongolia, China |
| Guowei Pan | Liaoning Provincial Center for Disease Control and Prevention, Liaoning, China |
| Guangzhi Pan | Liaoning Provincial Center for Disease Control and Prevention, Liaoning, China |
| Ling Jin | Liaoning Provincial Center for Disease Control and Prevention, Liaoning, China |
| Quanfu Yu | Liaoning Provincial Center for Disease Control and Prevention, Liaoning, China |
| Yunfei Liu | Liaoning Provincial Center for Disease Control and Prevention, Liaoning, China |
| Yingli Zhu | Jilin Provincial Center for Disease Control and Prevention, Jilin, China |
| Zhifang Cheng | Jilin Provincial Center for Disease Control and Prevention, Jilin, China |
| Jun Yang | Jilin Provincial Center for Disease Control and Prevention, Jilin, China |
| Chundi Jia | Jilin Provincial Center for Disease Control and Prevention, Jilin, China |
| Qing Wang | Jilin Provincial Center for Disease Control and Prevention, Jilin, China |
| Shichun Yan | Heilongjiang Provincial Center for Disease Control and Prevention, Heilongjiang, China |
| Jingyang Huang | Heilongjiang Provincial Center for Disease Control and Prevention, Heilongjiang, China |
| Yumei Chi | Heilongjiang Provincial Center for Disease Control and Prevention, Heilongjiang, China |
| Li Zhang | Heilongjiang Provincial Center for Disease Control and Prevention, Heilongjiang, China |
| Ruifa Pang | Heilongjiang Provincial Center for Disease Control and Prevention, Heilongjiang, China |
| Yan Shi | Shanghai Provincial Center for Disease Control and Prevention, Shanghai, China |
| Yan Lu | Shanghai Provincial Center for Disease Control and Prevention, Shanghai, China |
| Yiling Wu | Shanghai Provincial Center for Disease Control and Prevention, Shanghai, China |
| Jie Yu | Shanghai Provincial Center for Disease Control and Prevention, Shanghai, China |
| Chunxiang Wu | Shanghai Provincial Center for Disease Control and Prevention, Shanghai, China |
| Jinyi Zhou | Jiangsu Provincial Center for Disease Control and Prevention, Jiangsu, China |
| Yunxia Wu | Jiangsu Provincial Center for Disease Control and Prevention, Jiangsu, China |
| Ning Zhang | Jiangsu Provincial Center for Disease Control and Prevention, Jiangsu, China |
| Jing Sun | Jiangsu Provincial Center for Disease Control and Prevention, Jiangsu, China |
| Xiumei Tian | Jiangsu Provincial Center for Disease Control and Prevention, Jiangsu, China |
| Jieming Zhong | Zhejiang Provincial Center for Disease Control and Prevention, Zhejiang, China |
| Jingying Chen | Zhejiang Provincial Center for Disease Control and Prevention, Zhejiang, China |
| Kaixu Xie | Zhejiang Provincial Center for Disease Control and Prevention, Zhejiang, China |
| Lingjuan Fu | Zhejiang Provincial Center for Disease Control and Prevention, Zhejiang, China |
| Xiaohua Wang | Zhejiang Provincial Center for Disease Control and Prevention, Zhejiang, China |
| Yeji Chen | Anhui Provincial Center for Disease Control and Prevention, Anhui, China |
| Ling Zeng | Anhui Provincial Center for Disease Control and Prevention, Anhui, China |
| Zhenqian Cao | Anhui Provincial Center for Disease Control and Prevention, Anhui, China |
| Maomin Yang | Anhui Provincial Center for Disease Control and Prevention, Anhui, China |
| Biao Hu | Anhui Provincial Center for Disease Control and Prevention, Anhui, China |
| Wenling Zhong | Fujian Provincial Center for Disease Control and Prevention, Fujian, China |
| Qingyan Liu | Fujian Provincial Center for Disease Control and Prevention, Fujian, China |
| Zhiding Huang | Fujian Provincial Center for Disease Control and Prevention, Fujian, China |
| Qingbin Lai | Fujian Provincial Center for Disease Control and Prevention, Fujian, China |
| Yuyu Zhang | Fujian Provincial Center for Disease Control and Prevention, Fujian, China |
| Liping Zhu | Jiangxi Provincial Center for Disease Control and Prevention, Jiangxi, China |
| Yujing Zhao | Jiangxi Provincial Center for Disease Control and Prevention, Jiangxi, China |
| Wuming Tao | Jiangxi Provincial Center for Disease Control and Prevention, Jiangxi, China |
| Yonghai Tu | Jiangxi Provincial Center for Disease Control and Prevention, Jiangxi, China |
| Lin Zhou | Jiangxi Provincial Center for Disease Control and Prevention, Jiangxi, China |
| Xiaolei Guo | Shandong Provincial Center for Disease Control and Prevention, Shandong, China |
| Benzheng Chai | Shandong Provincial Center for Disease Control and Prevention, Shandong, China |
| Liping Ding | Shandong Provincial Center for Disease Control and Prevention, Shandong, China |
| Yuewei Zou | Shandong Provincial Center for Disease Control and Prevention, Shandong, China |
| Dongzhi Li | Shandong Provincial Center for Disease Control and Prevention, Shandong, China |
| Li Hua | Shandong Provincial Center for Disease Control and Prevention, Shandong, China |
| Shixian Feng | Henan Provincial Center for Disease Control and Prevention, Henan, China |
| Jianli Guo | Henan Provincial Center for Disease Control and Prevention, Henan, China |
| Wanguang Liu | Henan Provincial Center for Disease Control and Prevention, Henan, China |
| Qingxiang Li | Henan Provincial Center for Disease Control and Prevention, Henan, China |
| Yunzhi Zheng | Henan Provincial Center for Disease Control and Prevention, Henan, China |
| Minli Xu | Henan Provincial Center for Disease Control and Prevention, Henan, China |
| Lan Zhang | Hubei Provincial Center for Disease Control and Prevention, Hubei, China |
| Chunbo Li | Hubei Provincial Center for Disease Control and Prevention, Hubei, China |
| Yingchao Wan | Hubei Provincial Center for Disease Control and Prevention, Hubei, China |
| Chi Hu | Hubei Provincial Center for Disease Control and Prevention, Hubei, China |
| Shengping Xu | Hubei Provincial Center for Disease Control and Prevention, Hubei, China |
| Biyun Chen | Hunan Provincial Center for Disease Control and Prevention, Hunan, China |
| Huilin Liu | Hunan Provincial Center for Disease Control and Prevention, Hunan, China |
| Xiaojia Bian | Hunan Provincial Center for Disease Control and Prevention, Hunan, China |
| Wenwei Chen | Hunan Provincial Center for Disease Control and Prevention, Hunan, China |
| Honghua Li | Hunan Provincial Center for Disease Control and Prevention, Hunan, China |
| Yanjun Xu | Guangdong Provincial Center for Disease Control and Prevention, Guangdong, China |
| Fan Weng | Guangdong Provincial Center for Disease Control and Prevention, Guangdong, China |
| Hong Wu | Guangdong Provincial Center for Disease Control and Prevention, Guangdong, China |
| Weijin Zhang | Guangdong Provincial Center for Disease Control and Prevention, Guangdong, China |
| Liankai Zhu | Guangdong Provincial Center for Disease Control and Prevention, Guangdong, China |
| Jun Meng | Guangxi Provincial Center for Disease Control and Prevention, Guangxi, China |
| Jin Yang | Guangxi Provincial Center for Disease Control and Prevention, Guangxi, China |
| Yongsong Zeng | Guangxi Provincial Center for Disease Control and Prevention, Guangxi, China |
| Gaoke Zhai | Guangxi Provincial Center for Disease Control and Prevention, Guangxi, China |
| Lingfeng Qin | Guangxi Provincial Center for Disease Control and Prevention, Guangxi, China |
| Yi Lu | Guangxi Provincial Center for Disease Control and Prevention, Guangxi, China |
| Zhenwang Fu | Hainan Provincial Center for Disease Control and Prevention, Hainan, China |
| Shukuan Wu | Hainan Provincial Center for Disease Control and Prevention, Hainan, China |
| Caigang Li | Hainan Provincial Center for Disease Control and Prevention, Hainan, China |
| Na Wang | Hainan Provincial Center for Disease Control and Prevention, Hainan, China |
| Lianfen Chen | Hainan Provincial Center for Disease Control and Prevention, Hainan, China |
| Xianbin Ding | Chongqing Provincial Center for Disease Control and Prevention, Chongqing, China |
| Qi Zhou | Chongqing Provincial Center for Disease Control and Prevention, Chongqing, China |
| Yiling Xie | Chongqing Provincial Center for Disease Control and Prevention, Chongqing, China |
| Rui Chen | Chongqing Provincial Center for Disease Control and Prevention, Chongqing, China |
| Dongsheng Yang | Chongqing Provincial Center for Disease Control and Prevention, Chongqing, China |
| Ying Deng | Sichuan Provincial Center for Disease Control and Prevention, Sichuan, China |
| Youping Hu | Sichuan Provincial Center for Disease Control and Prevention, Sichuan, China |
| Bo Huang | Sichuan Provincial Center for Disease Control and Prevention, Sichuan, China |
| Ping Wang | Sichuan Provincial Center for Disease Control and Prevention, Sichuan, China |
| Peng Cai | Sichuan Provincial Center for Disease Control and Prevention, Sichuan, China |
| Changyan Peng | Sichuan Provincial Center for Disease Control and Prevention, Sichuan, China |
| Xiaofang Chen | Sichuan Provincial Center for Disease Control and Prevention, Sichuan, China |
| Ling Li | Guizhou Provincial Center for Disease Control and Prevention, Guizhou, China |
| Liang Xiao | Guizhou Provincial Center for Disease Control and Prevention, Guizhou, China |
| Chenglin Wang | Guizhou Provincial Center for Disease Control and Prevention, Guizhou, China |
| Chaogang Tan | Guizhou Provincial Center for Disease Control and Prevention, Guizhou, China |
| Daobin Chen | Guizhou Provincial Center for Disease Control and Prevention, Guizhou, China |
| Min Chen | Guizhou Provincial Center for Disease Control and Prevention, Guizhou, China |
| Mingfang Qin | Yunnan Provincial Center for Disease Control and Prevention, Yunnan, China |
| Zhaomin Lei | Yunnan Provincial Center for Disease Control and Prevention, Yunnan, China |
| Jianzhi Qiu | Yunnan Provincial Center for Disease Control and Prevention, Yunnan, China |
| Wei Yu | Yunnan Provincial Center for Disease Control and Prevention, Yunnan, China |
| Yunfei Li | Yunnan Provincial Center for Disease Control and Prevention, Yunnan, China |
| Guoxia Bai | Tibet Provincial Center for Disease Control and Prevention, Tibet, China |
| Ying Wang | Tibet Provincial Center for Disease Control and Prevention, Tibet, China |
| Lin Qiu | Shaanxi Provincial Center for Disease Control and Prevention, Shaanxi, China |
| Ning Wang | Shaanxi Provincial Center for Disease Control and Prevention, Shaanxi, China |
| Xudong Zhao | Shaanxi Provincial Center for Disease Control and Prevention, Shaanxi, China |
| Ani Yan | Shaanxi Provincial Center for Disease Control and Prevention, Shaanxi, China |
| Jianfei Liu | Shaanxi Provincial Center for Disease Control and Prevention, Shaanxi, China |
| Tingcai Wang | Gansu Provincial Center for Disease Control and Prevention, Gansu, China |
| Kaihua Hao | Gansu Provincial Center for Disease Control and Prevention, Gansu, China |
| Zaihong Zhang | Gansu Provincial Center for Disease Control and Prevention, Gansu, China |
| Zhanqi Xian | Gansu Provincial Center for Disease Control and Prevention, Gansu, China |
| Tenghuan Ma | Gansu Provincial Center for Disease Control and Prevention, Gansu, China |
| Zhihua Xu | Qinghai Provincial Center for Disease Control and Prevention, Qinghai, China |
| Yi Yang | Ningxia Provincial Center for Disease Control and Prevention, Ningxia, China |
| Hongli Wang | Ningxia Provincial Center for Disease Control and Prevention, Ningxia, China |
| Rong Li | Ningxia Provincial Center for Disease Control and Prevention, Ningxia, China |
| Zhonggang Zhao | Ningxia Provincial Center for Disease Control and Prevention, Ningxia, China |
| Caixia Fan | Ningxia Provincial Center for Disease Control and Prevention, Ningxia, China |
| Purhati | Xinjiang Provincial Center for Disease Control and Prevention, Xinjiang, China |
| Hongjun Xu | Xinjiang Provincial Center for Disease Control and Prevention, Xinjiang, China |
| Liping Gao | Xinjiang Provincial Center for Disease Control and Prevention, Xinjiang, China |
